# The relationship between empowerment and stigma among the Chinese people with diabetes: the mediating role of self-management and the moderating role of psychological resilience

**DOI:** 10.3389/fpsyg.2025.1591565

**Published:** 2026-01-02

**Authors:** Yujin Mei, Lin Zhang, Fuying Zhang, Yalin Shen, Mingjia Chen, Hui Guo

**Affiliations:** 1 The First Affiliated Hospital of Zhejiang Chinese Medical University (Zhejiang Provincial Hospital of Chinese Medicine), Hangzhou, Zhejiang, China; 2 Department of Internal Medicine Nursing, School of Nursing, Wannan Medical College, Wuhu, Anhui, China; 3 School of Nursing, Wannan Medical College, Wuhu, Anhui, China

**Keywords:** empowerment, people with diabetes, psychological resilience, self-management, stigma

## Abstract

**Background:**

Diabetes mellitus is highly prevalent in China, and individuals affected by this condition often experience stigma, which negatively impacts their psychological well-being and disease management. Empowerment interventions have the potential to reduce stigma by enhancing patients’ knowledge and skills; however, the mechanisms underlying this effect are not yet fully understood. The present study aimed to examine the mediating role of self-management behaviors and the moderating effect of psychological resilience (PR) in this context.

**Method:**

This study employed a cross-sectional design and included 329 individuals with diabetes, selected through a multistage stratified sampling method. Data collection instruments comprised the diabetes empowerment scale, the Connor-Davidson resilience scale, the stigma scale for chronic illness, and the diabetes management self-efficacy scale. Statistical analyses were performed using SPSS version 23.0, alongside the PROCESS macro, to investigate mediating and moderated mediation effects.

**Results:**

Empowerment (*r* = −0.451, *p* < 0.001), self-management (*r* = −0.397, *p* < 0.001), and PR (*r* = −0.325, *p* < 0.001) each demonstrated significant negative correlations with stigma. Furthermore, self-management was found to partially mediate the association between empowerment and stigma, accounting for 45.44% of the total effect [*β* = −0.169, 95% CI (−0.272, −0.088)]. PR significantly moderated the relationship between empowerment and self-management (*β* = 0.002, *p* < 0.001), as well as the direct relationship between empowerment and stigma (*β* = −0.012, *p* < 0.001). Simple slope analyses revealed that the positive influence of empowerment on self-management and the negative influence of empowerment on stigma were both more pronounced among individuals exhibiting higher levels of PR.

**Conclusion:**

Our study demonstrates that empowerment has a direct impact on the stigma experienced by individuals with diabetes, as well as an indirect effect mediated by self-management, with PR acting as a moderating factor. These findings suggest that healthcare practitioners should prioritize the enhancement of empowerment-based education and implement tailored interventions that consider patients’ varying levels of PR, in order to reduce stigma and promote better mental health outcomes.

## 1 Introduction

Diabetes mellitus (DM) has emerged as one of the most rapidly increasing chronic diseases on a global scale. The International Diabetes Federation (IDF) reported that approximately 537 million individuals were affected by diabetes in 2021, with projections indicating this figure will surpass 783 million by 2045 (Hossain et al., 2024). In China, the total number of individuals with diabetes has reached 141 million, the highest worldwide, corresponding to an adult prevalence rate of approximately 12.8% (Ji et al., 2024). DM and its associated complications impose significant health and economic burdens on both patients and society, impacting diverse age groups, particularly middle-aged and elderly populations (Gregg et al., 2018; Goswami, 2024).

Diabetes-related stigma encompasses the experiences and perceptions of rejection, social isolation, labeling, discrimination, and devaluation encountered by individuals with diabetes as a consequence of their condition. This stigma frequently engenders feelings of self-blame, shame, guilt, and a diminished sense of self-worth (Rai et al., 2020; Pillen and Ward, 2022; Guo et al., 2023). IDF has recognized diabetes stigma as a pressing concern warranting immediate intervention (Speight et al., 2024). A comprehensive survey conducted across 17 countries, involving 8,596 patients, revealed that approximately 19.2% of respondents reported instances of discrimination (Foryciarz et al., 2016). Within the Chinese cultural milieu, diabetes is often erroneously attributed to personal failings such as laziness, overeating, or lack of self-discipline (Davidsen et al., 2022). Moreover, the condition may be regarded as a source of familial dishonor or a hereditary defect, leading patients to conceal their diagnosis (Schabert et al., 2013; Kaur and Sinha, 2024). Healthcare providers may inadvertently contribute to stigma by characterizing patients as uncooperative or noncompliant, with such judgments intensifying the patients’ stigma experiences (Hill, 2010; Litterbach et al., 2024). Empirical evidence indicates that individuals who have encountered diabetes-related stigma exhibit a threefold increase in the risk of depression and anxiety relative to the general population (Robinson et al., 2023). The apprehension of external judgment stemming from stigma can also deter patients from monitoring their blood glucose levels or administering insulin in public settings (Ashraf and Cheng, 2024). Further research has demonstrated a significant association between stigma and suboptimal glycemic control, as well as an elevated risk of diabetes-related complications (Akyirem et al., 2023).

Empowerment is defined as the process through which individuals with diabetes augment their sense of control over both their condition and their lives by acquiring knowledge, developing skills, and gaining access to relevant resources, this process facilitates their transition from passive recipients to active decision-makers (Vainauskienė and Vaitkienė, 2021; Ugliara et al., 2021). Empirical studies have demonstrated a significant negative correlation between empowerment and stigma (Omodara et al., 2022). Empowerment can directly alleviate the effects of stigma by fostering patients’ autonomy, enhancing their knowledge, and improving their psychological well-being (Lanfredi et al., 2015). Knowledge empowerment enables patients to overcome cognitive biases and attains a more accurate understanding of the disease, thereby diminishing uncritical acceptance of the self-responsibility paradigm (Joo et al., 2020; Ozpamuk et al., 2023). Skill empowerment bolsters patients’ confidence in self-management practices, allowing them to navigate daily diabetes care with greater ease and reducing stigma associated with procedural errors (Natale et al., 2023). Furthermore, psychological empowerment supports patients in reconstructing their self-identity, encouraging the transformation of their illness experiences into adaptive coping strategies and mitigating feelings of stigma (Moda et al., 2025).

Self-management is defined as the capacity of patients to sustain disease stability through proactive monitoring, behavioral adjustments, and emotional regulation, with an emphasis on autonomy and continuity (Davis et al., 2022). According to social cognitive theory (Bandura, 1989), there exists a triadic reciprocal interaction among personal factors, behavior, and the environment. Within this framework, empowerment, an individual cognitive construct, exerts influence on self-management behaviors, which in turn can modify an patients’ perception of their social environment. Studies have demonstrated a bidirectional and mutually reinforcing relationship between empowerment and self-management in the context of diabetes prevention and management (Funnell and Anderson, 2004). Empowerment enhances patients’ autonomy and perceived self-efficacy, thereby fostering intrinsic motivation and facilitating external support for self-management activities. Conversely, effective self-management reinforces the sense of empowerment through positive experiential feedback (Wang et al., 2022; Hickmann et al., 2022). Moreover, empowerment aids patients in comprehending disease mechanisms and treatment rationales, mitigating fear and maladaptive behaviors, and restructuring belief systems to improve self-management outcomes (Náfrádi et al., 2017; Ting et al., 2025). Additionally, proficient self-management has been identified as a factor that mitigates patients’ experiences of stigma (Inagaki et al., 2022; Othman et al., 2022; van Olmen, 2022). Stigma, characterized as a covert negative emotional experience, can undermine self-confidence; however, effective self-management can bolster patients’ confidence in managing their condition, thereby attenuating the impact of stigma (Wang et al., 2022). Furthermore, to circumvent stigma arising from social discrimination, patients are required to cultivate effective self-management practices (Le Forestier et al., 2022). Collectively, these findings suggest that self-management is influenced by empowerment and concurrently affects stigma. Accordingly, we propose the hypothesis that self-management serves as a mediating variable in the relationship between empowerment and stigma among individuals with diabetes.

PR is widely acknowledged as a beneficial psychological characteristic, defined as an individual’s capacity to adapt effectively in the aftermath of adverse experiences, particularly those that are traumatic, challenging, or pose threats to life and health (Fletcher and Sarkar, 2013). Drawing on the transactional theory of stress and coping (Lazarus and Folkman, 1984), the influence of stressors, such as perceived stigma, is not direct but is mediated through an individual’s appraisal process and the resources available to them. Empirical evidence (Mei et al., 2023; Zhao et al., 2022) indicates that individuals with diabetes who exhibit higher levels of PR are capable of transforming negative experiences into sources of motivational resilience, thereby substantially mitigating the effects of stigma encountered during discrimination. Moreover, patients with elevated PR demonstrate greater proficiency in translating empowerment-based education into actionable behaviors, which facilitates improved acceptance of their condition (Zhang et al., 2023). For instance, research has revealed that following instruction on diabetes dietary guidelines, patients with higher PR achieved a compliance rate that was 58% greater than that of patients with lower PR (Blaique et al., 2023). When faced with treatment decisions, individuals with stronger PR are better equipped to evaluate the benefits and risks, enabling them to undertake manageable risks rather than avoid decision-making due to fear (Dubois et al., 2020). Additionally, robust PR enhances rational cognitive processes, allowing patients to adapt more effectively to their current health status and environment, thereby improving their self-management capabilities (Padamsee et al., 2023). Given its role as a critical psychological resource for managing disease-related stress and sustaining health-promoting behaviors, it is hypothesized that PR may serve as a moderating factor in the relationship among empowerment, self-management, and stigma in individuals with diabetes.

**Figure 1:**
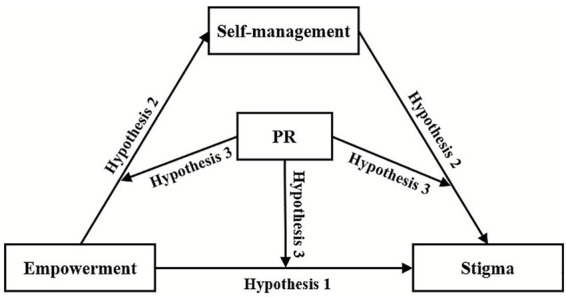
A hypothesized model illustrating the relationship between empowerment, self-management, PR, and stigma. Hypothesis 1: Empowerment is a significant negative predictor of stigma among individuals with diabetes. Hypothesis 2: Self-management mediates the relationship between empowerment and stigma in individuals with diabetes. Hypothesis 3: PR moderates the relationships among empowerment, self-management, and stigma in individuals with diabetes. PR, psychological resilience.

In summary, while preliminary investigations have examined the relationships among empowerment, self-management, and stigma, and the protective function of PR has been recognized, the combined effects of these three factors on patients’ experiences of stigma remain insufficiently understood. Accordingly, informed by social cognitive theory, the transactional theory of stress and coping, and pertinent empirical studies, we have developed a moderated mediation model (Figure 1) to elucidate the complex interactive mechanisms among these variables in a novel and innovative manner. This model offers a theoretical basis for the development of targeted psychological intervention strategies for patients. Based on this framework, we advance the following hypotheses:

Hypothesis 1: Empowerment is a significant negative predictor of stigma among individuals with diabetes.

Hypothesis 2: Self-management mediates the relationship between empowerment and stigma in individuals with diabetes.

Hypothesis 3: PR moderates the relationships among empowerment, self-management, and stigma in individuals with diabetes.

## 2 Materials and methods

### 2.1 Participants

From June to September 2022, this study employed a multistage stratified sampling method to collect data. In the initial stage, at the hospital level, simple random sampling was employed to select the First Affiliated Hospital of Wannan Medical College from the pool of all tertiary hospitals. Subsequently, at the departmental level, simple random sampling was again applied to choose the Endocrinology and Geriatrics Departments within the selected hospital. At the patient level, survey sites were established in both outpatient and inpatient units of these departments, where eligible patients were recruited through systematic random sampling. Inclusion criteria encompassed: (i) patients meeting the diagnostic standards for diabetes mellitus as defined by the American Diabetes Association (2010); (ii) patients who were conscious and exhibited full mobility and cognitive function; and (iii) patients willing to participate and complete the questionnaire. Exclusion criteria included: (i) severe mental impairment or intellectual disabilities; (ii) advanced diabetes complications or inability to self-care; (iii) presence of other serious conditions such as severe cardiovascular disease, infectious diseases, cancer, or sensory impairments attributable to diabetes complications; and (iv) pregnancy or other specific diabetes types. According to the criteria proposed by Kendall (2004), a sample size of at least 10 times the number of items (with an additional 10% buffer) is recommended. Among the scales used in this study, the diabetes empowerment scale (DES) had the highest number of items (28 items). Therefore, the base sample size was calculated as 280 cases. Considering the 10% buffer, the required sample size was 308 cases. A total of 334 questionnaires were distributed, with 329 usable questionnaires returned, yielding a valid response rate of 98.5%.

### 2.2 Data collection

This study was approved by the Ethics Committee of Wannan Medical College prior to its initiation (Approval Number: 2021-3). All patients were fully informed of the study’s purpose, content, and privacy protection measures before the survey and provided written informed consent. All collected data were encrypted and stored securely, with access restricted to authorized researchers only. Additionally, all scales used in this study were obtained with prior authorization from the original developers. To minimize errors, researchers received standardized training before the survey to clarify communication techniques and scoring criteria. This cross-sectional observational study strictly adhered to the Strengthening the Reporting of Observational Studies in Epidemiology (STROBE) guidelines to ensure rigor and transparency in reporting. All research procedures were conducted in accordance with the principles of the Declaration of Helsinki.

### 2.3 Measurements

#### 2.3.1 Diabetes empowerment scale

The diabetes empowerment scale (DES) was developed by Anderson et al. (2000). The scale consists of three dimensions: psychosocial management of diabetes, assessment of dissatisfaction and readiness for change, and setting and achieving diabetes goals, comprising a total of 28 items. A 5-point Likert scale was used, ranging from 0 (strongly disagree) to 4 (strongly agree). Higher scores indicate greater empowerment. The Cronbach’s alpha for the scale in this study was 0.960 (Zhu et al., 2019).

#### 2.3.2 Diabetes self-management scale

The diabetes management self-efficacy scale (DMSES), initially developed in 1999, comprises 20 items that reflect the tasks a patient must perform to manage this condition (Bijl et al., 1999). The scale encompasses four dimensions: blood glucose monitoring, foot examination, physical exercise and weight management, and medical care. It employs an 11-point scale ranging from 0 (cannot do at all) to 10 (certainly can do). Higher scores indicate greater self-management efficacy. In this study, the scale demonstrated a Cronbach’s alpha of 0.890 (Hurst et al., 2022).

#### 2.3.3 Connor-Davidson resilience scale

The Connor-Davidson Resilience Scale (CD-RISC) was developed by psychologists Professors Connor and Davidson (2003). The CD-RISC comprises 25 items rated on a five-point Likert scale ranging from 0 (not at all true) to 4 (almost always true). The scale assesses five dimensions: high standards, resilience, and ability; emotional regulation and trust in one’s intuition; a constructive attitude towards change and secure relationships; perceived control; and mental strength. Higher scores indicate greater PR. In this study, the scale demonstrated good internal consistency, with a Cronbach’s alpha of 0.861 (Fu et al., 2014).

#### 2.3.4 Stigma scale for chronic illness

Rao et al. (2009) developed the stigma scale for chronic illness (SSCI) to measure stigma in individuals with chronic illnesses. The scale comprises 24 items and includes two dimensions: intrinsic stigma and extrinsic stigma. The first 13 items assess internal stigma by inquiring about the respondent’s feelings of stigma. The remaining 11 items evaluate stigma experienced due to external actions. Each item is rated on a scale from 0 (never) to 4 (always), with higher scores indicating more frequent experiences of stigma. In this study, the scale demonstrated good reliability, with a Cronbach’s alpha of 0.829 (Lu et al., 2019).

### 2.4 Data analysis

All statistical analyses were conducted using SPSS 23.0. Initially, we confirmed that the data were normally distributed by assessing their skewness and kurtosis. Subsequently, descriptive statistics were employed to examine the general characteristics of the patients. Pearson correlation coefficient was utilized to perform bivariate correlation analyses, preliminarily verifying the direction and strength of the relationships between variables, thereby providing a foundation for constructing the mediation model. Finally, after including relevant control variables (complications and diabetes education), we applied Model 4 of the PROCESS macro in SPSS 23.0 to evaluate the mediating effect of stigma, and guided by a strong *a priori* theoretical foundation and the study’s hypothesized framework, we selected Model 59 to comprehensively test the moderating role of PR, in accordance with the principles of theory-driven research design. All continuous variables were standardized, and interaction terms were computed based on these standardized scores. The 95% confidence intervals (CIs) for the mediating and moderating effects were generated using the bootstrap method with 5,000 resamples. Effects were considered significant if the 95% CIs did not include zero.

## 3 Results

### 3.1 Descriptive statistics

Table 1 presents the demographic characteristics of the patients alongside the univariate analysis of morbidity stigma scores according to various factors. Of the 329 patients, 198 (60.2%) were male and 131 (39.8%) were female. Ages ranged from 45 to 95 years. There was a statistically significant difference (*p* < 0.05) in stigma scores with respect to medical insurance, complications, glucose monitoring, diet plan, diabetes education, and hypoglycemia. Analysis revealed that only 8.5% of patients were able to perform regular and periodic blood glucose monitoring. More than 37.4% of patients had a monthly income below 1,000 CNY, with the vast majority struggling to make ends meet. Additionally, 68.7% of patients had complications, while 70.5% had not received any diabetes-related education.

**Table 1:** Univariate analysis of stigma with different characteristics (*n* = 329).

Variables	Group	*N* (%)	Mean ± SD	*F/t*	*p*
Gender	Male	198 (60.2)	33.08 ± 16.01	0.245	0.621
	Female	131 (39.8)	34.31 ± 17.00		
Monthly income	<1,000 CNY	123 (37.4)	38.24 ± 17.09	2.018	0.111
	1,000 ~ 3,000 CNY	55 (16.7)	32.31 ± 16.38		
	3,000 ~ 5,000 CNY	77 (23.4)	37.30 ± 14.81		
	Above 5,000 CNY	74 (22.5)	34.66 ± 16.41		
Medical insurance	Publicly insurance	20 (6.1)	22.85 ± 15.65	6.712	<0.001
	Staff insurance	285 (86.6)	36.53 ± 16.02		
	Other insurance	22 (6.7)	44.55 ± 16.06		
	No insurance	2 (0.6)	34.00 ± 2.83		
Years of illness	<5 Years	101 (30.7)	37.98 ± 17.63	0.559	0.642
	5 ~ 10 Years	86 (26.1)	35.55 ± 16.06		
	11 ~ 20 Years	93 (28.3)	35.41 ± 15.09		
	>20 Years	49 (14.9)	35.33 ± 16.87	8.545	<0.001
Complication	Yes	103 (31.3)	41.73 ± 17.41		
	No	226 (68.7)	33.77 ± 15.35		
Glucose monitoring	Never	106 (32.2)	41.22 ± 14.98	13.657	<0.001
	Irregular	195 (59.3)	35.22 ± 16.12		
	Regular	28 (8.5)	24.32 ± 16.41		
Diet plan	Full implemented	25 (7.5)	15.76 ± 14.71	26.035	<0.001
	Mostly implemented	115 (35.0)	32.34 ± 14.48		
	Partially implemented	97 (29.5)	41.13 ± 15.30		
	No implemented	92 (28.0)	41.46 ± 14.62		
Diabetes education	Yes	97 (29.5)	42.86 ± 17.18	24.138	<0.001
	No	232 (70.5)	33.45 ± 15.24		
Hypoglycemia	Yes	78 (23.7)	31.12 ± 15.40	10.209	<0.001
	No	251 (76.3)	37.81 ± 16.39		

SD, standard deviation.

### 3.2 Bivariate correlation analysis

A correlation analysis was conducted on empowerment, self-management, PR, and stigma among individuals with diabetes. The mean values, standard deviations, and correlation coefficients for each variable are presented in Table 2. The stigma score was 36.22 ± 16.39, indicating a high level of stigma among patients in this study. The empowerment score was 37.18 ± 13.16, suggesting a moderately low level of empowerment. The self-management and PR scores were 17.28 ± 5.91 and 33.73 ± 13.71, respectively, indicating that both self-management and PR were at relatively low levels. The results of the correlation analysis showed that empowerment (*r* = −0.451, *p* < 0.001), self-management (*r* = −0.397, *p* < 0.001), and PR (*r* = −0.325, *p* < 0.001) were all negatively correlated with stigma (Hypothesis 1). Additionally, empowerment was positively correlated with self-management (*r* = 0.381, p < 0.001) and PR (*r* = 0.434, *p* < 0.001). Self-management (*r* = 0.398, *p* < 0.001) was positively correlated with PR.

**Table 2:** Descriptive statistics and correlations among variables (*n* = 329).

Variables	Mean	SD	Empowerment	Self-management	PR	Stigma
Empowerment	37.18	13.16	−			
Self-management	17.28	5.91	0.381***	−		
PR	33.73	13.71	0.434***	0.398***	−	
Stigma	36.22	16.39	−0.451***	−0.397***	−0.325***	−

Correlation analysis using Pearson’s correlation coefficient.

***p < 0.001; PR, psychological resilience; SD, standard deviation.

### 3.3 Mediation analysis

To verify the mediating role of self-management in the relationship between empowerment and stigma (Hypothesis 2), regression analyses were conducted controlling for two demographic variables, namely complication and diabetes education. The mediating effect of self-management on the relationship between empowerment and stigma was examined in SPSS using the PROCESS macro (Model 4) proposed by Hayes. The results of the analyses are presented in Table 3. The findings showed that in the mediation model, empowerment remained positively associated with self-management (*β* = 0.176, *p* < 0.001). Both empowerment (*β* = −0.203, *p* < 0.001) and self-management (*β* = −0.096, *p* < 0.001) were negatively associated with stigma. With the addition of the mediating variable self-management, the *F*-value of the model increased from 29.633 to 51.611, representing a 74.2% increase in the model’s explanatory power. The indirect [*β* = −0.169, *SE* = 0.047, 95% CI = (−0.272, −0.088)] and direct [*β* = −0.203, *SE* = 0.066, 95% CI = (−0.333, −0.073)] effects of empowerment on stigma through self-management were also tested and are shown in Table 4. The results indicate that the indirect effect accounts for 45.44% of the total effect, calculated by dividing the indirect effect (−0.169) by the total effect (−0.372). The significance of the indirect path was tested using bootstrapping with 5,000 resamples, yielding a confidence interval that does not include zero, while the direct effect accounts for 54.56%. Furthermore, when self-management was included as a mediating variable in the model, the direct effect of empowerment on stigma was attenuated compared to the total effect, indicating that self-management played a partial mediating role. These results suggest that empowerment not only directly reduces stigma but also indirectly reduces stigma by enhancing patients’ self-management capabilities.

**Table 3:** Testing the mediation effect of empowerment on stigma.

Variables	Self-management				Stigma			
	*β*	*SE*	*t*	95% CI	*β*	*SE*	*t*	95% CI
Complication	−1.697	0.563	−3.012^**^	−2.805, −0.588	4.782	1.648	2.902^**^	1.540, 8.025
Diabetes education	2.478	0.287	−6.977^**^	1.914, 3.042	−4.260	0.918	4.641^**^	−6.065, −2.454
Empowerment	0.176	0.021	8.516^***^	0.136, 0.217	−0.203	0.066	−3.073^***^	−0.333, −0.073
Self-management					−0.096	0.160	−5.997^***^	−1.275, −0.645
*R^2^*			0.268				0.323	
*F*			29.633				51.611	

**p < 0.01; ***p < 0.001; CI, confidence interval; *SE*, standard error.

**Table 4:** Effects of empowerment on stigma with self-management as a mediator.

Effect type	Effects	BootSE	BootLLCI	BootULCI	Relative effect size (%)
Indirect effect	−0.169	0.047	−0.272	−0.088	45.44%
Direct effect	−0.203	0.066	−0.333	−0.073	54.56%
Total effect	−0.372	0.063	−0.358	−0.090	100%

LLCI, lower level of confidence interval; ULCI, lower level of confidence interval.

### 3.4 The moderation analyses

The mediating role of self-management between empowerment and stigma has been previously validated. Therefore, to examine the moderating role of PR in this mediating model of empowerment, self-management, and stigma, we controlled for two demographic variables, namely complications and diabetes education. The moderating role of PR (Hypothesis 3) was tested in SPSS using the PROCESS macro (Model 59) developed by Hayes. Specifically, we constructed two models: Model 1 assessed the moderating effect of PR on the pathway from empowerment to self-management, while Model 2 evaluated the moderating effects of PR on both the empowerment-to-stigma and self-management-to-stigma pathways.

**Figure 2:**
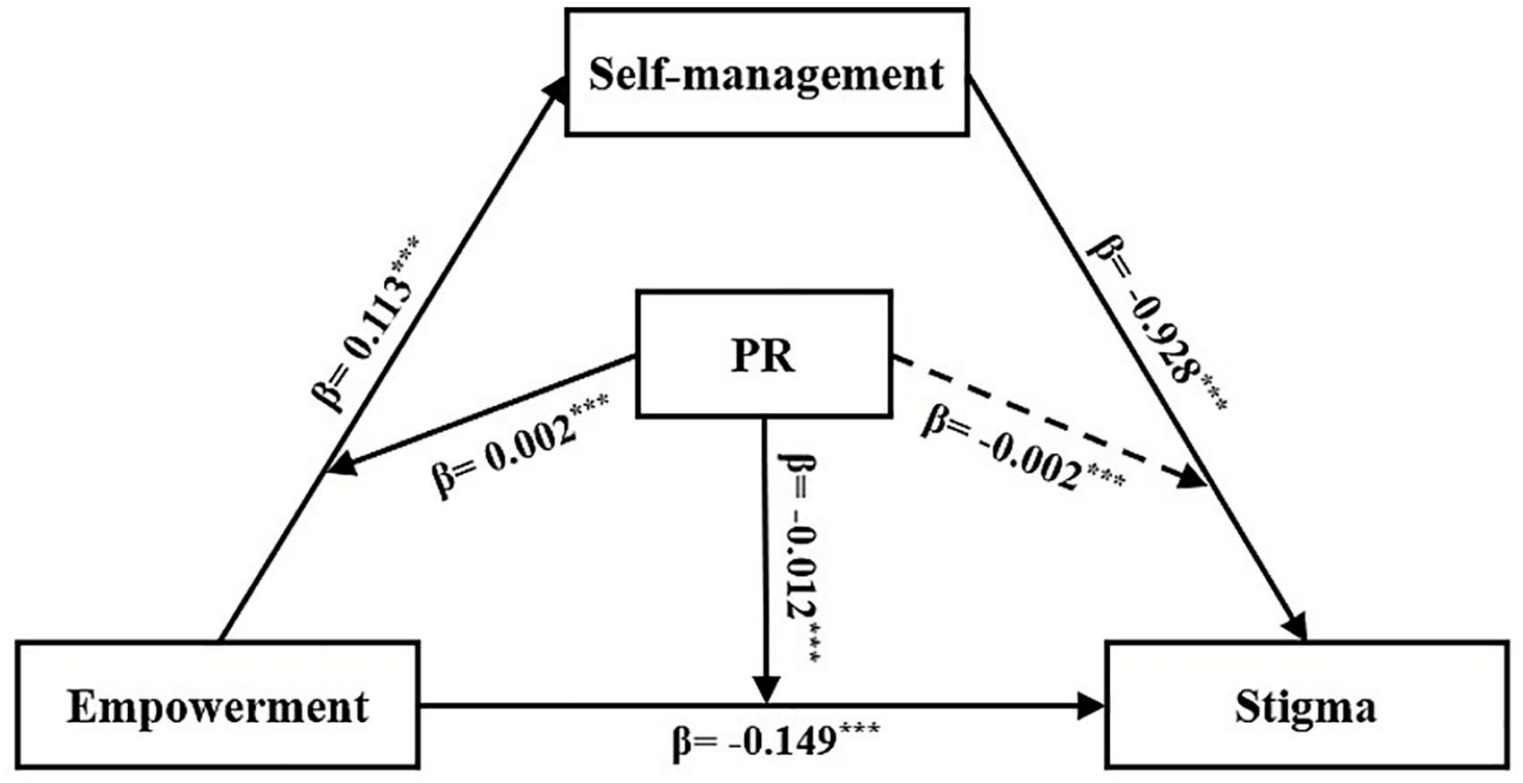
The moderated mediation model. ***p < 0.001. The solid lines represented PR having a moderating effect, while the dotted line represented PR not having a moderating effect; PR, psychological resilience.

The validation results are presented in Table 5. Empowerment [β* = 0.252, *β* = 0.113, *SE* = 0.028, 95% CI = (0.058, 0.168)] and PR [β* = 0.263, *β* = 0.114, *SE* = 0.023, 95% CI = (0.068, 0.160)] remained positively associated with self-management in Model 1. Additionally, PR acted as a moderator between empowerment and self-management [β* = 0.066, *β* = 0.002, *SE* = 0.001, 95% CI = (0.000, 0.004)]. In Model 2, empowerment [β* = −0.337, *β* = −0.419, *SE* = 0.073, 95% CI = (−0.563, −0.276)], self-management [β* = −0.335, *β* = −0.928, *SE* = 0.145, 95% CI = (−1.214, −0.643)], and PR [β* = −0.286, *β* = −0.342, *SE* = 0.065, 95% CI = (−0.471, −0.214)] were all negatively associated with stigma. PR moderated only the relationship between empowerment and stigma [β* = −0.133, *β* = −0.012, *SE* = 0.003, 95% CI = (−0.018, −0.006)], but not between self-management and stigma [*β* = −0.002, *SE* = 0.007, 95% CI = (−0.017, 0.013)]. The final mediation model is illustrated in Figure 2. The *R^2^* value indicates the proportion of variance in the dependent variable explained by the model. For Model 1, *R^2^* = 0.243 (*F* = 20.743, *p* < 0.001), demonstrating that this model accounts for 24.3% of the variation in self-management. Model 2 yielded *R^2^* = 0.364 (*F* = 26.206, *p* < 0.001), indicating that this model accounts for 36.4% of the variance in stigma. The inclusion of interaction terms significantly increased *R^2^*, confirming the incremental validity of the models.

**Table 5:** Results of the moderated mediation model analysis.

Variables	Model 1 (Self-management)						Model 2 (Stigma)					
	β*	*β*	*SE*	*t*	LLCI	ULCI	β*	*β*	*SE*	*t*	LLCI	ULCI
Complication		−1.351	0.61	−2.214**	−2.551	−0.150		2.658	1.567	1.696**	0.426	5.742
Diabetes education		0.917	0.675	1.359*	0.411	2.244		−6.802	1.725	−3.944*	−10.196	−3.409
Empowerment	0.252	0.113	0.028	4.052***	0.058	0.168	−0.337	−0.419	0.073	−5.740***	−0.563	−0.276
Self-management							−0.335	−0.928	0.145	−6.401***	−1.214	−0.643
PR	0.263	0.114	0.023	4.858***	0.068	0.160	−0.286	−0.342	0.065	−5.248***	−0.471	−0.214
Empowerment × PR	0.066	0.002	0.001	1.927***	0.000	0.004	−0.133	−0.012	0.003	−4.019***	−0.018	−0.006
Self-management × PR								−0.002	0.007	−0.265	−0.017	0.013
*R^2^*				0.243						0.364		
*F*				20.743						26.206		

*p < 0.05, **p < 0.01, ***p < 0.001. PR, psychological resilience; *β*, unstandardized coefficient; β*, standardized coefficient; *SE*, standard error; LLCI, lower level of confidence interval; ULCI, lower level of confidence interval.

**Figure 3:**
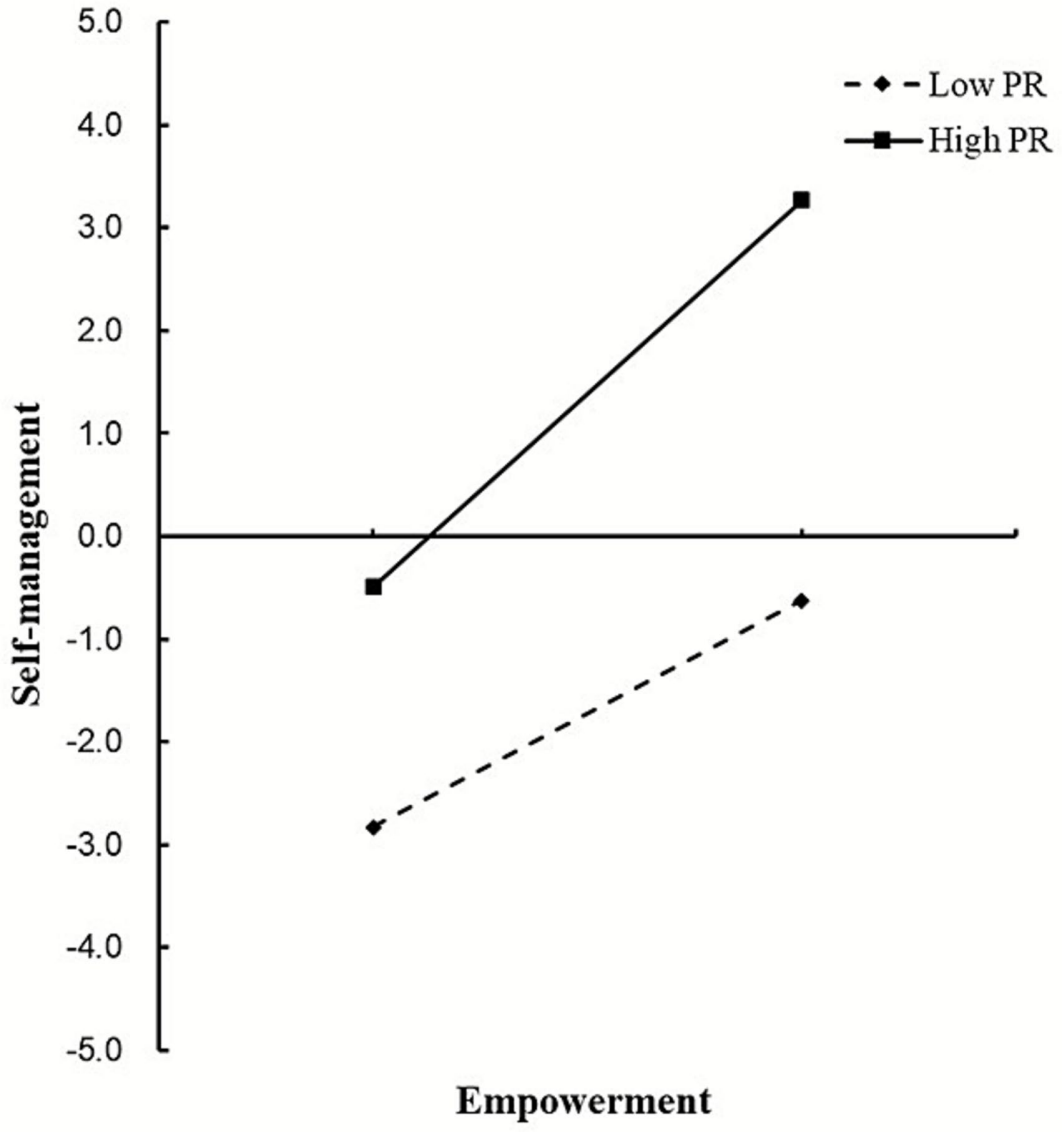
The moderating role of PR in the relationship between empowerment and self-management. PR, psychological resilience.

Figure 3 offers a more intuitive illustration of how PR moderates the effect of empowerment on self-management. Simple slope tests indicate that patients with higher PR demonstrate significantly greater levels of self-management compared to those with lower PR. Furthermore, as empowerment levels increase, self-management also shows an upward trend, with this improvement being more pronounced among patients with higher PR.

**Figure 4:**
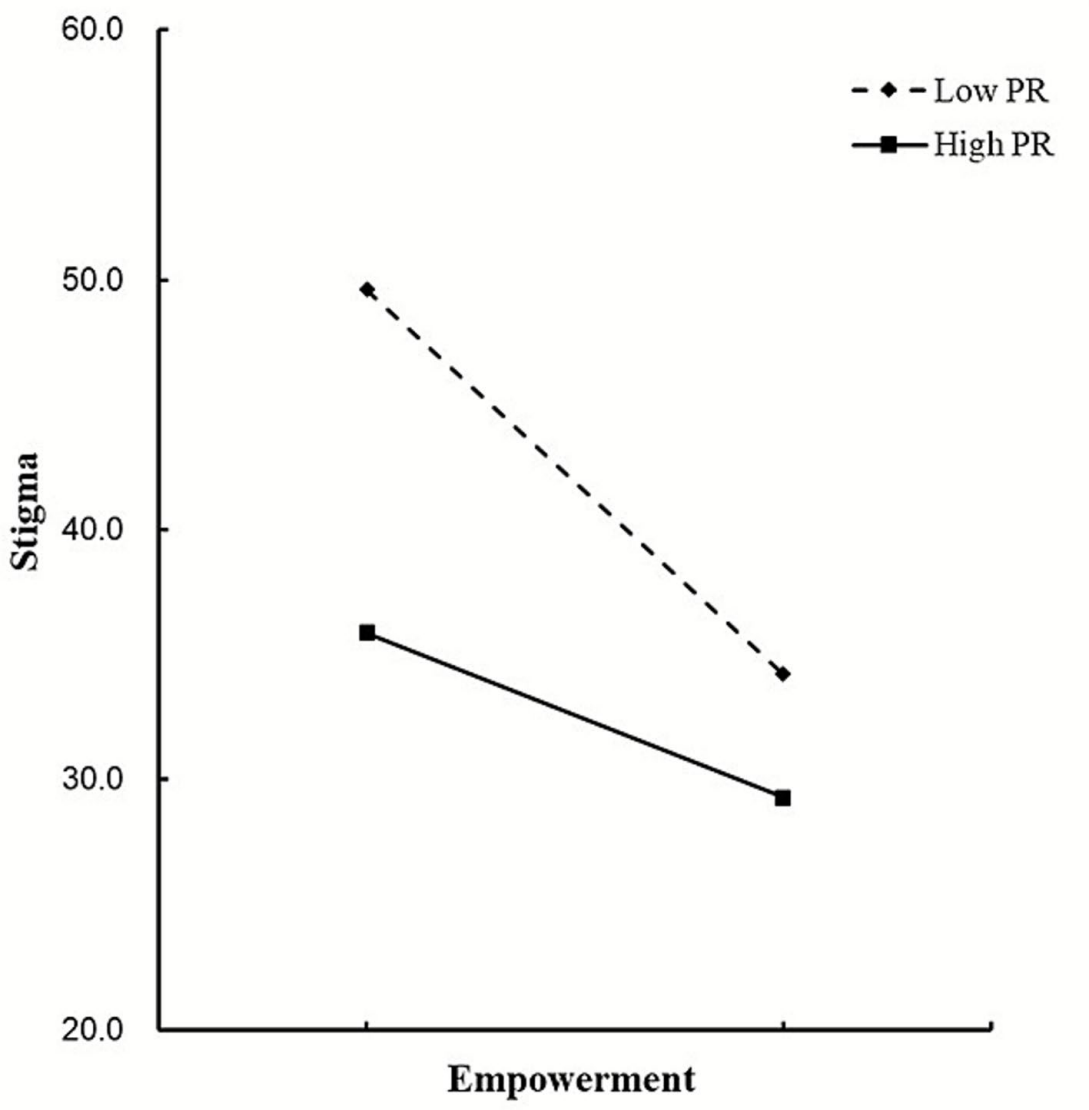
The moderating role of PR in the relationship between empowerment and stigma. PR, psychological resilience.

Figure 4 shows how PR moderates the relationship between empowerment and stigma. A simple slope test revealed that patients with high PR had a significantly lower initial level of stigma compared to those with low PR. Furthermore, as the level of empowerment increased, the level of stigma in patients exhibited a decreasing trend, with the reduction in stigma being more pronounced in patients with high PR than in those with low PR.

## 4 Discussion

In this study, a moderated mediation model was developed to demonstrate that empowerment influences stigma through self-management in individuals with diabetes. PR moderated both the empowerment and self-management components of the mediation model, as well as the empowerment–stigma relationship. Moderation analyses revealed that empowerment had a significant effect on self-management and stigma when individuals with diabetes exhibited high levels of PR. Furthermore, patients with high PR showed a greater increase in self-management and a more pronounced decrease in stigma compared to those with low PR.

In this study, we found that empowerment continued to have a significant negative predictive effect on patients’ stigma after controlling for other variables. The higher the level of empowerment, the lower the level of stigma experienced by patients. Meanwhile, a previous study (Puhl et al., 2020) reported stigma scores for individuals with diabetes of 29.24 ± 9.67; in our study, the score was 36.22 ± 16.39, which was slightly higher than that previously reported. Diabetes mellitus (DM) is a chronic disease that not only requires ongoing medical management but may also expose patients to discriminatory social treatment and high levels of disease-related stigma (Akugizibwe et al., 2023; Li et al., 2023). Appropriate empowerment education and an individual’s social adaptability are closely related to stigma in patients and play a crucial role in mitigating adverse outcomes, such as stigma, by positively influencing bodily function, subjective well-being, and improving patients’ quality of life (Shih, 2004; Kariyawasam et al., 2022).

Previous studies have shown that the negative impact of stigma on individuals with diabetes involves a key psychological mediating mechanism, such as self-esteem. However, the specific nature of this mechanism may vary depending on cultural emphases on individual agency, collective norms, or disease attribution (Ozturk et al., 2022). In this study, self-management serves as the principal mediating variable, indicating that the effect of empowerment on stigma among patients occurs through both a direct pathway and an indirect pathway that influences self-management. Research has confirmed that empowerment is a crucial factor in alleviating negative emotions and unhealthy behaviors among patients (Ho et al., 2010). If patients receive appropriate empowerment education following diagnosis, they are more likely to adopt a positive and optimistic attitude towards the disease, transforming stress into motivation, thereby reducing the experience of disease-related stigma and mitigating its adverse effects (Hickmann et al., 2022). Through empowerment tools such as knowledge and skills, patients can develop effective management behaviors, better control their condition, and improve their health (Chen et al., 2018). Patients who effectively manage their condition (for example, through regular diet and exercise) may reduce the social prejudice and discrimination they face, increase their engagement with the outside world, and consequently lower their levels of stigma (Cho et al., 2022; Akyirem et al., 2023). Therefore, to address the stigma experienced by individuals with diabetes, it is essential to focus on both the direct impact of empowerment on stigma and its indirect impact via self-management.

This study found that PR played a significant moderating role in the mediation model linking empowerment to self-management. For patients with higher levels of PR, empowerment had a substantial impact on self-management. The greater the patients’ level of PR, the stronger their understanding and control over their condition, and the more confident they were in implementing treatment plans and managing their condition (McKenna et al., 2022). Additionally, higher PR could reduce the burden of disease, protect patients’ physical and mental health, and promote the implementation of management plans (Wang et al., 2024; Logie et al., 2022). Furthermore, PR also moderated the direct impact of empowerment on stigma among patients. Specifically, those with higher PR experienced a greater reduction in stigma after receiving empowerment, indicating that they were more likely to experience a decrease in stigma as a result of this intervention. The study suggested that when enhancing patients’ sense of empowerment, attention should also be paid to their levels of PR.

Interestingly, this study found that the moderating effect of PR on the relationship between self-management and internalized stigma did not reach statistical significance (*P* > 0.05), indicating that PR did not significantly moderate the association between self-management and stigma. This finding suggests that the impact of self-management on reducing mental health stigma may operate through a relatively robust and direct behavioral–cognitive pathway, with mechanisms less dependent on individuals’ internal emotion-regulation capacities or resilience-related resources (Niu et al., 2025). Furthermore, other studies have reported that individuals participating in “self-management recovery training” showed significantly reduced levels of internalized stigma, and this effect was independent of protective factors such as resilience and self-esteem, further highlighting the potential of self-management as a “direct intervention effect” (Yimer et al., 2025; Wang et al., 2023). Therefore, the non-significant moderation observed in the present study may imply that self-management interventions possess broad applicability in reducing individual-level stigma, a possibility that warrants further investigation in future research. Moreover, patients in this study exhibited relatively low overall levels of self-management (17.28 ± 5.91), and more than 70.5% had not received diabetes-related education. This likely indicates a lack of awareness among patients regarding the association between self-management and stigma, which may further diminish the moderating role of psychological resilience (PR). These issues warrant further investigation in future research.

Our findings provide valuable insights. This study introduces a validated moderated mediation model that elucidates the psychological mechanisms through which empowerment reduces stigma among individuals with diabetes in China. It emphasizes the critical roles of self-management and PR, suggesting that future interventions and theoretical frameworks should consider these factors synergistically. In clinical practice, our results advocate a multifaceted approach to mitigating diabetes-related stigma. Healthcare providers should move beyond traditional didactic education towards empowerment-oriented strategies that enhance patients’ autonomy, knowledge, and skills. Based on our findings, we also recommend that healthcare providers adopt differentiated intervention strategies in clinical practice. For patients with higher baseline levels of PR, clinical interventions can focus on strengthening and refining their self-management skills—for example, by setting more complex health goals. In contrast, for patients with lower baseline PR, interventions should prioritize enhancing resilience, and then gradually introduce self-management education on this foundation, thereby more precisely and effectively reducing their perceived stigma.

## 5 Limitations

This study has several limitations. First, as it employed a cross-sectional design, we were unable to draw causal inferences from the observed associations. Future research should utilize longitudinal studies to further refine the pathways in our theoretical model. Second, although self-reporting is widely used in the literature, it has inherent drawbacks, including a high degree of subjectivity, which inevitably introduces bias into the data. Future studies should incorporate multiple data collection methods to enable cross-validation and obtain more objective and accurate data. Third, this study was conducted at a single center, which may limit the generalizability of our findings to the broader Chinese population with diabetes. Future multi-center studies are warranted to validate our results across diverse healthcare settings. Finally, only one influencing factor, PR, was included in this study; additional factors could be incorporated in future research.

## 6 Conclusion

This study confirmed that empowerment influences patients’ stigma through both direct effects and indirect effects mediated by self-management, with PR playing a moderating role in this process. These findings reveal the complex psychological and behavioral mechanisms underlying the stigma experienced by patients and suggest that healthcare professionals should not only priorities empowerment education for patients but also integrate PR stratification with the core strategy of enhancing self-management to more effectively improve stigma and disease management outcomes among Chinese patients.

## Data Availability

The raw data supporting the conclusions of this article will be made available by the authors, without undue reservation.
